# Gut-on-a-Chip for Infectious Diseases: A Pathogen-Centric Review of Model Selection and Validation

**DOI:** 10.34133/bmef.0336

**Published:** 2026-11-09

**Authors:** Shujun Nie, Zhifeng Li, Zilin Zhang, Boyang Song, Jun Ouyang, Yuan Xu, Huadong Zhang, Zaozao Chen, Zhongze Gu, Wanjiang Gu

**Affiliations:** ^1^ Chongqing Key Laboratory of Highly Pathogenic Microbes, Chongqing Research Center for Disease Prevention and Public Health, Chongqing Center for Disease Control and Prevention (Chongqing Academy of Preventive Medicine), Chongqing 400707, China.; ^2^ State Key Laboratory of Digital Medical Engineering, School of Biological Science and Medical Engineering, Southeast University, Nanjing 210096, Jiangsu, China.; ^3^ Jiangsu Avatarget Biotechnology Co. Ltd., Suzhou 215153, Jiangsu, China.

## Abstract

Gut-on-a-chip systems are microfluidic, 3-dimensional (3D) in vitro platforms designed to recapitulate selected intestinal features, including epithelial polarity, fluid flow, peristalsis-like deformation, mucus formation, oxygen gradients, and, in some designs, epithelial–endothelial or host–microbiome interactions. Rather than treating increasing structural complexity as an inherent surrogate for biological fidelity, this review evaluates gut-on-a-chip models according to the infectious-disease question being addressed, the specific biological functions reproduced, the measurable infection endpoints, and the level of experimental or translational validation. We synthesize evidence across bacterial, viral, and parasitic infections and compare the relative maturity of these applications. Bacterial models currently represent the most mature evidence base, whereas fungal infections are considered where direct gut-on-a-chip evidence remains limited. We further examine host–microbiome interactions and gut–organ-axis platforms only when they provide a mechanistic or translational perspective relevant to infection. Four major evidence gaps are identified: limited head-to-head benchmarking against established models or clinical observations, incomplete standardization of chip and infection parameters, limited culture duration and microbial complexity in many systems, and relatively sparse infection-specific applications of artificial intelligence. Finally, we propose a validation-oriented framework linking pathogen, biological question, required chip feature, measurable endpoint, and evidence level, and we clarify the current regulatory position of new approach methodologies (NAMs).

## Introduction

### Gut-on-a-chip: Definition and technological essence

A gut-on-a-chip is a microfluidic, 3-dimensional (3D) biomimetic system designed to recapitulate selected mechanical and biochemical features of the intestinal microenvironment [1,2]. Core features may include villus-like architecture, controlled mechanical forces and fluid shear stress (FSS), coculture of intestinal epithelial and endothelial cells, and dynamic integration of microbial communities [3–6].

Regulatory interest in new approach methodologies (NAMs) has increased, but regulatory encouragement should not be interpreted as broad qualification of gut-on-a-chip systems as replacements for animal studies. In the United States, the U.S. Food and Drug Administration (FDA) has promoted the development and regulatory use of NAMs [7–9] and, in March 2026, issued draft guidance describing general principles for their validation and use in drug development [10]; this guidance is nonbinding and is not specific to gut-on-a-chip systems. The European Commission’s 2025 European Chemicals Industry Action Plan is a policy communication for the chemical sector rather than a legally binding qualification framework for organ-on-chip technologies [11]. In parallel, China has also issued GB/T 44831-2024 as a national technical standard for skin-on-a-chip [12], while related guidelines from the Center for Drug Evaluation (CDE) of the National Medical Products Administration (NMPA) mention organoid and organ-chip data among nonclinical research inputs. Accordingly, this review treats regulatory activity as an enabling policy context while emphasizing that fit-for-purpose validation and context-specific regulatory acceptance remain necessary.

### The need and evolution for advanced intestinal infection models

Intestinal infections caused by bacteria (e.g., Salmonella), viruses [e.g., severe acute respiratory syndrome coronavirus 2 (SARS-CoV-2)], and parasites (e.g., Cryptosporidium) impose a substantial global health burden and can contribute to persistent intestinal inflammation and other disease sequelae [13,14]. The World Health Organization identifies diarrheal disease as a major global health burden [15], underscoring the need for physiologically relevant models to study pathogenesis and therapeutic interventions, including infections caused by emerging viruses such as SARS-CoV-2 with gastrointestinal manifestations [16].

The past decade has witnessed substantial advances in microfluidics, microfabrication, organoid culture, 3D bioprinting, and genome editing [17,18]. However, this evolution should not be interpreted as a simple hierarchy in which each newer model is universally more predictive than its predecessor. For infectious-disease studies, model selection should instead be matched to pathogen tropism, route of exposure, mucus and mechanical requirements, immune or vascular interactions, and the endpoint of interest. The central question is therefore not whether a platform is structurally more complex, but whether the added complexity generates information that is relevant to the biological or translational question under study. Recent syntheses published in 2025–2026 reinforce the need to distinguish model complexity from demonstrated validity [19–21].

### Scope, literature strategy, and conceptual contribution

This review is a focused narrative synthesis rather than a formal systematic review or meta-analysis. We prioritized primary studies that directly interrogated intestinal infection, host–pathogen interactions, host–microbiome interactions relevant to infection, or infection-relevant gut–organ communication, together with recent high-impact reviews that define the broader organ-chip landscape. Recent reviews published in 2025–2026 further indicate that the field is expanding, while standardization and validation remain unresolved [20–25]. Table 1 summarizes the key characteristics of the included reviews. Therefore, the primary contribution of this review is not to catalogue available gut-on-a-chip technologies, but to provide an evidence-based framework for determining when and why these platforms are informative for infectious-disease research.

**Table 1.** Comparison of the present review with recent reviews of organ-on-chip, intestinal organ chips, and host–microbiome models

Review	Primary scope	Infection emphasis	Gut/gut–organ focus	Validation/standards	Distinct conceptual contribution of this review
Alonso-Roman et al. [23]	Infectious-disease organ-on-chip models	Broad, multi-organ	Gut is one application	General opportunities and limitations	Centers intestinal infection and uses a pathogen-to-validation framework
Özkan et al. [24]	Intestinal organ chips for disease modeling	Infection within broader disease scope	Strong intestinal focus	Disease modeling and translation	Compares infection maturity across pathogen classes
Shin et al. [25]	Host–microbiome organ-on-chip	Not infection-centered	Broad microbiome focus	Engineering and microbiome complexity	Links microbial ecology to infection phenotypes and reproducibility
Chai et al. [22]	Gut-on-chip fabrication, models, microbiome and axes	Infection is one application	Explicit gut–organ-axis coverage	Highlights standardization barriers	Adds infection-specific evidence maturity and validation readiness
The present review	Gut-on-a-chip for infectious diseases	Pathogen-centered	Gut-centric; axes only when infection-relevant	Separates biomimicry from predictive validity	Pathogen → question → chip feature → endpoint → validation level → limitation

## Technological Advances in Gut-on-a-Chip Engineering

### Cell composition and culture systems: A historical progression

In vitro intestinal models have evolved from standardized 2D monolayers [26–28] to organoid-derived cultures [29] and microphysiological gut-on-a-chip systems [30,31]. These models can partly address some limitations associated with species differences in traditional animal models [32,33]. The integration of advanced methodologies, such as real-time cell imaging, multi-omics analysis, and physiological and biochemical monitoring, has facilitated research into host–microbiome interactions, antibiotic sensitivity profiling, and drug metabolism (Fig. 1). However, the relevant comparison for infection research is functional rather than chronological. Caco-2 and related cocultures remain useful for high-throughput adhesion, invasion, barrier-disruption, and transport assays. Organoids and organoid-derived monolayers provide broader epithelial diversity and patient- or species-specific phenotypes, while chip systems add controlled flow, mechanical deformation, compartmentalization, and opportunities for immune or microbial integration (Table 2). The added complexity is most informative when it changes an infection-relevant endpoint that is poorly represented in a simpler model. Recent work on infection microenvironments similarly emphasizes that organoids, planar cultures, and microphysiological systems address different experimental questions rather than forming a universal hierarchy [19,20].

**Fig. 1.**
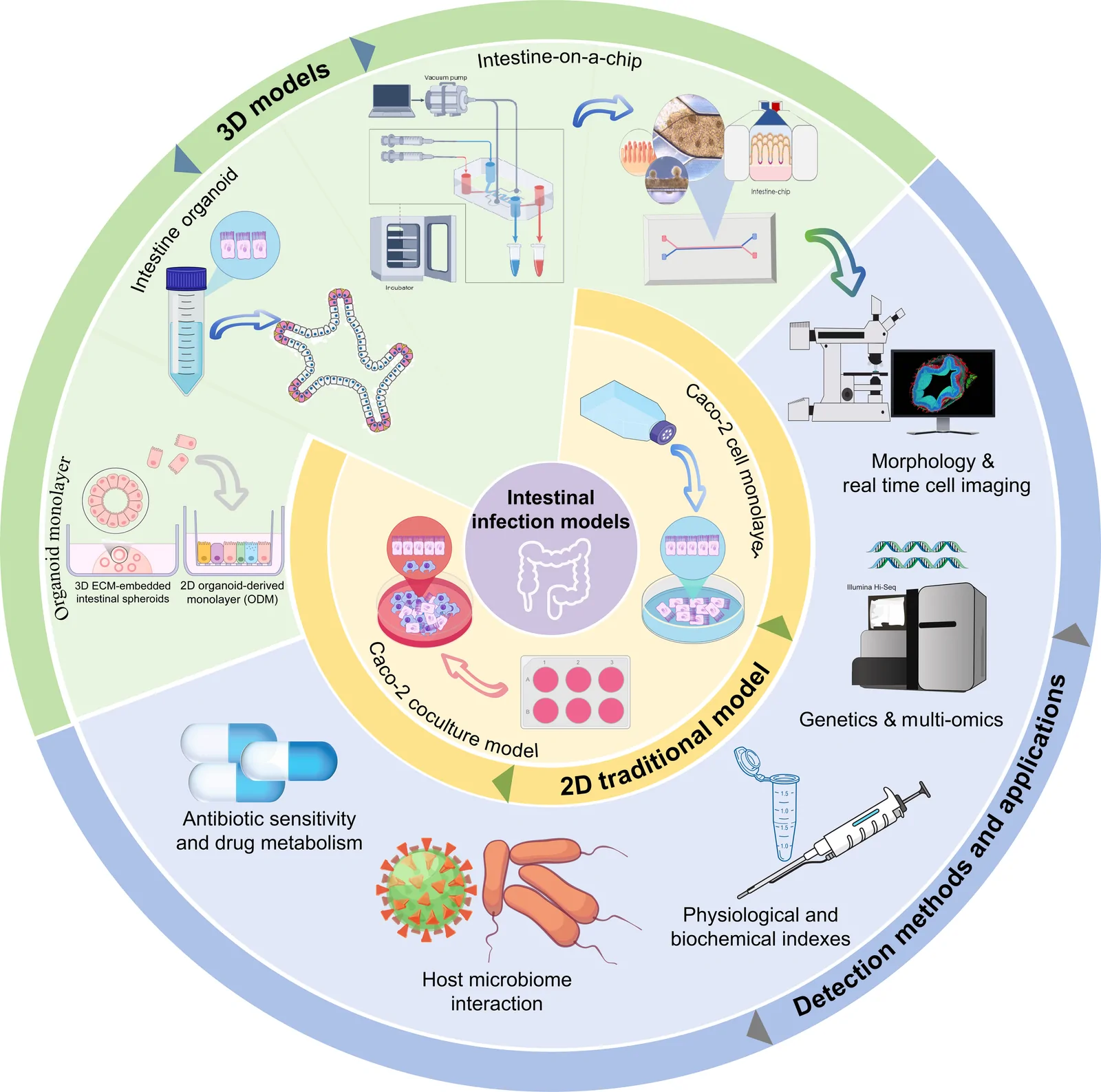
Evolution of intestinal infection models toward question-driven validation. The figure summarizes conventional 2D models, including Caco-2 monolayers and coculture models, and 3D models, including intestinal organoids, organoid-derived monolayers, and gut-on-a-chip systems. Created using WPS; some icons were drawn in-house; and the remaining icons were sourced from Bioicons (https://bioicons.com/) and SciDraw (https://scidraw.io/).

**Table 2.** Evidence-oriented progression of in vitro intestinal infection models

Model/stage	Representative milestone	Infection questions	Evidence-supported strengths	Major limitations
2D Caco-2 [27]	Differentiated epithelial monolayer	Adhesion, invasion, barrier injury, toxins	Low cost; high throughput; standardized assays	Cancer-derived background; limited mucus, diversity and mechanics
2D coculture [34]	Caco-2 plus mucus/immune companion cells	Mucus or epithelial–immune interactions	Adds selected host functions	Still simplified and model-dependent
3D intestinal organoid [29]	Stem-cell-derived self-organizing epithelium	Tropism, epithelial responses, regeneration	Cellular diversity; donor/species specificity	Closed lumen; incomplete vascular/mechanical context
Organoid-derived 2D [38,98]	Apically accessible organoid-derived monolayer	Direct infection and intervention studies	Combines organoid phenotype with access	Loses 3D geometry and dynamic mechanics
Basic gut-on-a-chip [30,31]	Perfused epithelial compartment	Colonization under flow; barrier/mechanical effects	Dynamic microenvironment; real-time monitoring	Cost, fabrication, and device variability
Complex gut-on-a-chip [40]	Endothelium, immune cells, microbiota and/or gradients	Host–pathogen–microbiome–immune interactions	Integrates multiple infection-relevant cues	High complexity; limited throughput, standardization and benchmarking

#### 2D models—the foundational era

The Caco-2 cell line remains a practical foundation for infection studies because differentiated monolayers provide polarized epithelial barriers and reproducible readouts for adhesion, invasion, permeability, and toxin-mediated injury [26–28]. Coculture systems, such as Caco-2/HT29-MTX or epithelial–immune combinations, can introduce selected mucus or inflammatory functions [34,35]. These models are therefore not obsolete; rather, their appropriate use is question-dependent. Their principal limitations are the cancer-derived background, incomplete cellular diversity, simplified mucus biology, and absence of physiological flow and tissue-scale mechanics [36,37].

#### 3D organoids—expanded cellular representation

The development of self-organizing intestinal organoids from Lgr5^+^ stem cells enabled culture of multiple epithelial lineages and donor- or species-specific phenotypes [29]. Organoids can support pathogen-specific studies in which epithelial differentiation, receptor expression, regenerative responses, or cell-type tropism are central. Organoid-derived monolayers further improve apical accessibility for infection experiments [38]. Nevertheless, closed luminal architecture, incomplete vascular and immune components, and lack of dynamic mechanical stimulation mean that organoids and organoid-derived monolayers answer different questions from perfused chips. Recent infection-model work also highlights culture-associated transcriptional drift as a potential confounder, underscoring the need to benchmark organoid phenotypes before interpreting pathogen-specific responses [39].

#### Gut-on-a-chip—integrated biomimicry and dynamic control

Gut-on-a-chip systems add controlled fluid flow, mechanical deformation, compartmentalization, and—depending on design—mucus, endothelial, immune, microbial, or oxygen-gradient modules [40,41]. The pioneering human gut-on-a-chip model developed by Kim and colleagues [30,31] introduced cyclic mechanical strain to mimic peristalsis and established an air–liquid interface. These features can be informative when infection phenotypes depend on colonization under flow, barrier mechanics, oxygen partitioning, or cross-compartment signaling. However, greater biomimicry should not be equated with demonstrated predictive superiority. The translational value of each added feature depends on whether it changes an infection-relevant endpoint and whether that endpoint has been benchmarked against established models or human observations.

Table 3 compares the model classes using evidence-supported capabilities and limitations rather than a single ordinal physiological-relevance score. This distinction is important because a model may have high structural fidelity for one feature while remaining insufficiently validated for another.

**Table 3.** Comparison of evidence-supported characteristics of in vitro intestinal infection models

Comparison indicator	2D models		3D organoid models		Organ-on-chip models	
	Caco-2 monolayer	Coculture model	Intestinal organoid	Organoid-derived 2D monolayer	Basic gut-on-a-chip	Complex gut-on-a-chip
Cell source	Cancer-derived human epithelial line	Caco-2 plus selected mucus/immune companion cells	Mouse or human stem-cell-derived intestinal cultures	Cells derived from intestinal organoids	Human cell line or primary/derived intestinal cells	Epithelial + endothelial and/or immune/microbial components
Spatial organization	2D polarized monolayer	2D with selected mucus/cell interactions	3D crypt-/villus-like organization	2D polarized monolayer	3D microfluidic compartment with controllable interfaces	3D multi-compartment microphysiological system
Dynamic physical cues	Limited	Limited	Absent or not physiologically controlled	Absent or limited	Flow and, in some devices, cyclic strain	Flow, strain, gradients, and additional modules depending on design
Typical infection readouts	Adhesion, invasion, TEER/permeability, cytotoxicity	Barrier, mucus-associated interactions, cytokines	Tropism, replication, epithelial injury, cell-state responses	Apical infection, replication, barrier changes	Colonization/invasion under flow, barrier and mechanical responses	Multi-compartment host–pathogen–immune–microbiome responses
Evidence-supported strengths	Standardization and throughput	Adds selected host factors	Cellular diversity and donor/species specificity	Accessible apical exposure with organoid phenotype	Dynamic microenvironment and real-time monitoring	Integration of multiple infection-relevant cues
Major limitations	Limited cellular diversity; cancer background	Model-dependent composition; limited mechanics	Closed lumen; limited vascular/mechanical context	Loss of 3D architecture	Cost, fabrication complexity, device-to-device variation	High complexity; reproducibility, throughput, and validation remain challenging
Validation status for infection prediction	Widely used mechanistic assay platform	Useful for targeted mechanistic questions	Growing pathogen-specific evidence	Useful for accessible infection assays	Increasing pathogen-specific evidence	Promising but comparatively sparse head-to-head or clinical benchmarking
References	[26,27,36,99]	[34–37]	[29,32,38,100]	[38,101]	[3,30,31,40–43]	[3,40,41,73,102–104]

### Fundamental models and design principles

For infection studies, chip architecture should be designed around the biological question rather than maximal complexity. Relevant design variables include cell source and differentiation state, extracellular matrix or membrane composition, channel geometry, perfusion regime, cyclic strain, oxygen distribution, mucus formation, and the location of microbial or immune compartments [24,25,42–44], as illustrated in Fig. 2. Controlled pathogen introduction, anaerobic modules, and real-time immune-cell imaging can then be added when required by the pathogen or endpoint [23,30,45–48].

**Fig. 2.**
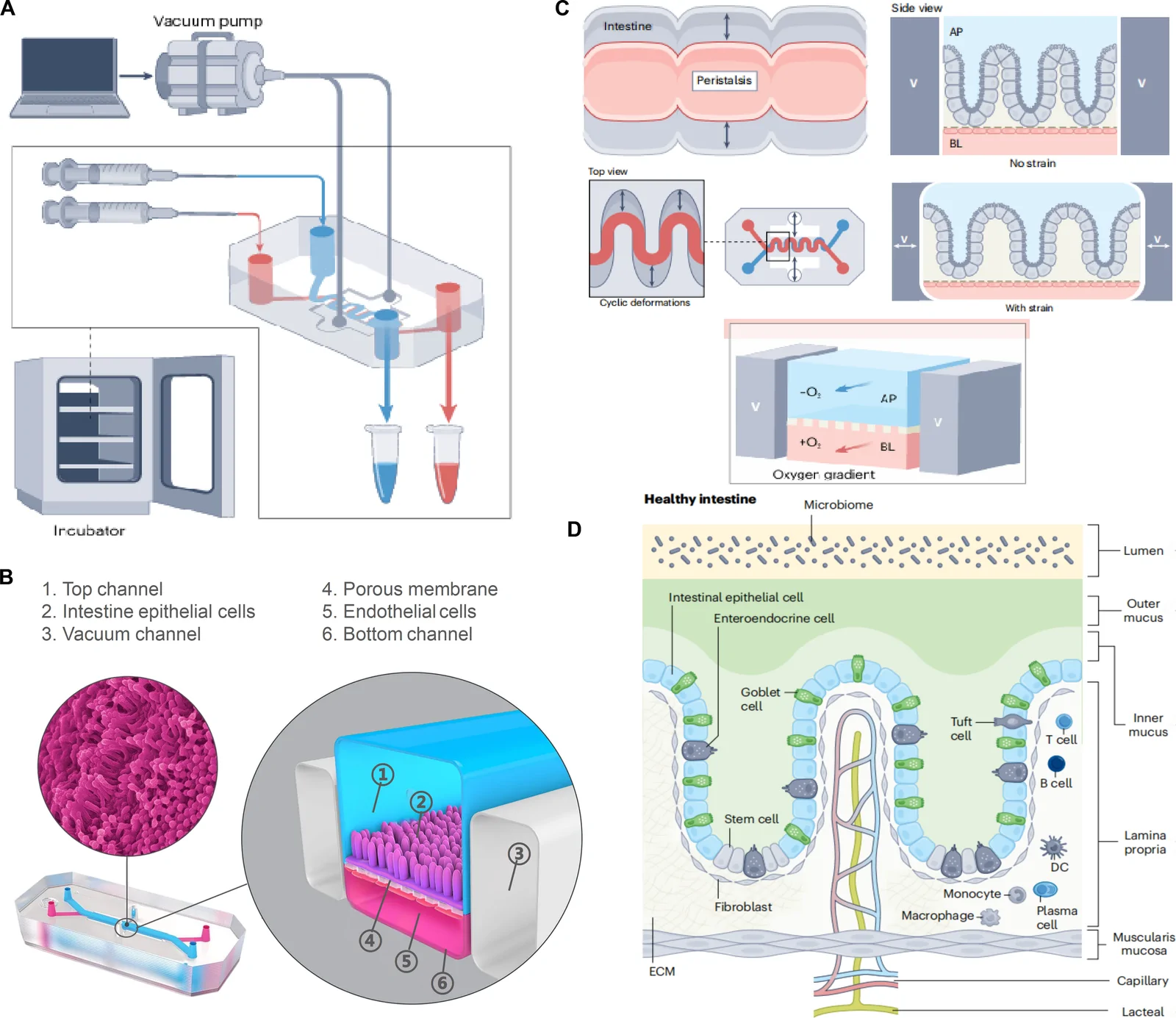
Gut-on-a-chip platform. (A) Equipment and configuration of a gut-on-a-chip model for host–microbiome coculture. A syringe pump controls the flow of culture medium at different volumetric flow rates, generating gradients of nutrients, biochemical molecules, oxygen, and fluid shear stress. Red and blue arrows indicate vascular and luminal flows, respectively. A computer-controlled actuation system applies vacuum-driven mechanical deformations during host–microbiome coculture. The experimental setup is maintained in a humidified CO_2_ incubator [25]. (B) Basic gut-on-a-chip architecture, including top and bottom channels, intestinal epithelial cells, endothelial cells, a porous membrane, and vacuum channels. Continuous microfluidic flow mimics luminal and vascular flow, supplies nutrients and exogenous molecules, and facilitates removal of microbial cells and their waste products [44]. (C) Intestinal peristalsis-like motion can be recreated by vacuum-driven cyclic actuation. Introduction of anoxic culture medium into the luminal microchannel can further establish a transmural oxygen gradient [25]. Cross-sectional views illustrate the applied cellular strain. AP, apical compartment; BL, basolateral compartment; V, vacuum chamber. (D) Basic structure of the healthy intestine [24]. The intestinal mucosal interface comprises stem, absorptive, goblet, epithelial, and other differentiated cells, together with a protective mucus layer, stromal cells such as fibroblasts, tissue-resident or circulating immune cells including dendritic cells (DCs), macrophages, lymphocytes (T cells and B cells) and neutrophils, and extracellular matrix (ECM).

Representative gut-on-a-chip models include static monolayer cultures derived from earlier organ-on-chip approaches [49], fluid-perfusion systems that established key principles for gut-on-a-chip development [30], epithelial–endothelial coculture models [40], commensal microbiome-integrated systems, and multi-organ chip (MOC) platforms, including gut–microbiota–neural axis [50] and brain–gut-axis models [51,52], as illustrated in Fig. 3. Notably, the Neurohumix chip, which cocultures neurons with gut microbiota, has provided evidence that microbial metabolites can modulate host immunity via the vagus nerve [50,52].

**Fig. 3.**
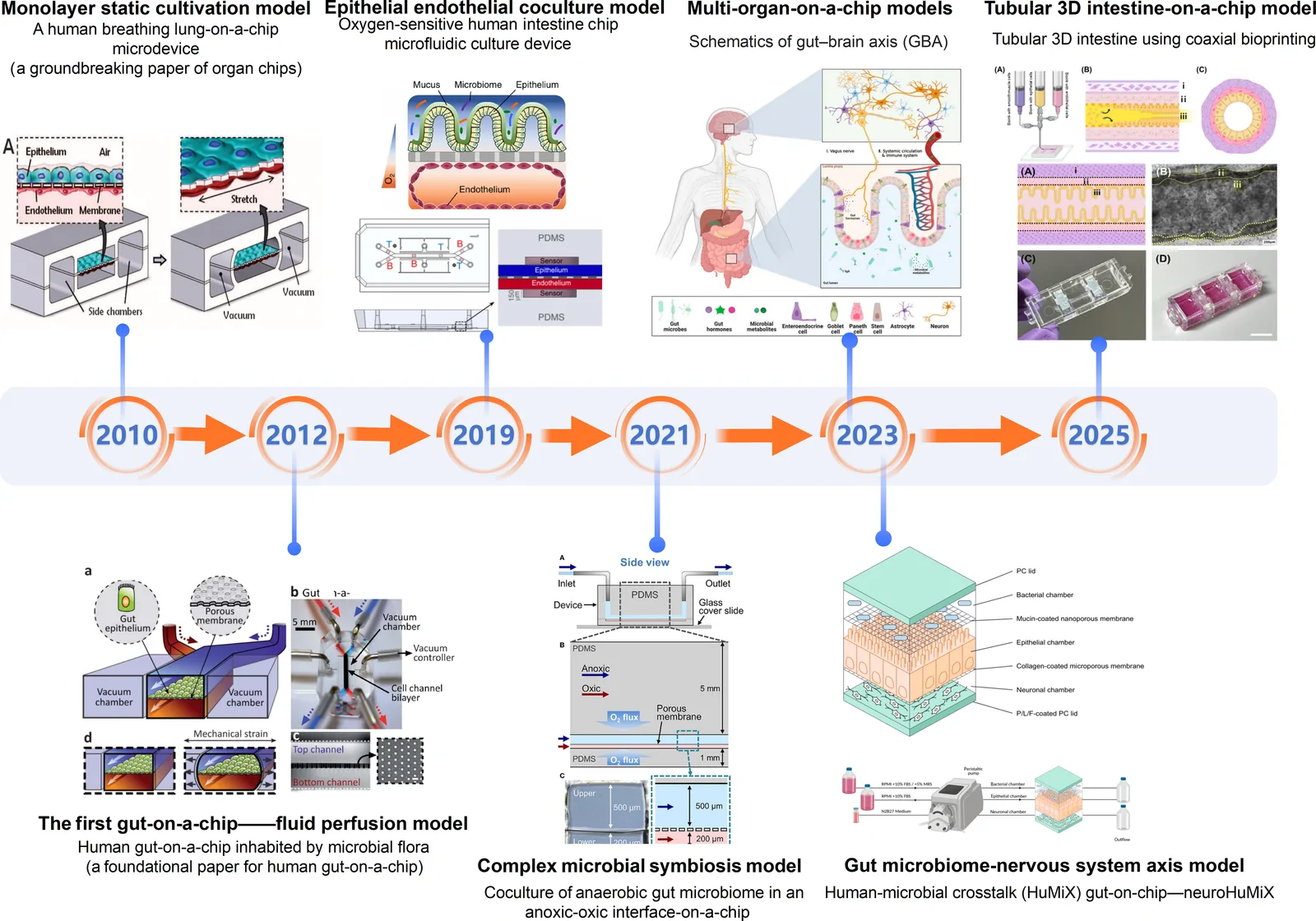
Representative gut-on-a-chip models [30,40,49–51,92,96].

### Simulation and regulation of the mechanical microenvironment

Dynamic mechanical conditions can alter epithelial differentiation, barrier function, mucus organization, and pathogen–surface interactions [30,48]. For infection research, the most informative comparisons are those in which a defined flow or strain condition changes a measurable phenotype—such as adhesion, invasion, barrier loss, immune recruitment, or microbial persistence—relative to a static control.

#### Mechanical stress

Peristalsis-like cyclic strain has been used to enhance epithelial differentiation and villus-like organization in chip cultures [30,48]. Such conditioning may improve the relevance of infection experiments when pathogen colonization or barrier responses depend on epithelial maturation. However, strain frequency and magnitude are device-specific and should be reported rather than treated as universal physiological values.

#### Fluid shear stress

FSS can alter mucus secretion and epithelial organization [30,53]. In infection studies, this is relevant because mucus thickness and turnover can modify pathogen access to epithelial receptors and influence colonization. Claims about downstream signaling or quantitative changes should therefore be interpreted in the context of the specific cell source, geometry, medium, and flow regime used in each study.

### Simulation and regulation of intestinal secretory functions

Secretory functions are most relevant to infection when they alter the chemical or physical niche encountered by pathogens, such as mucus, antimicrobial factors, bile acid exposure, or epithelial mediators. Here, we focus on intestinal mucus secretion. Other secretory modules, including enteroendocrine hormones and digestive enzymes, are useful for broader physiological modeling but currently have a more limited direct role in the infection-centered literature.

Mucus is a key infection-relevant interface because it regulates pathogen diffusion, receptor access, and interactions with commensal microorganisms [40,53–55]. Flow and cell composition can alter mucus organization and MUC2 expression [53,56,57] (Fig. 4). For cross-study comparison, investigators should report the mucus-producing cell source, mucus thickness or composition, flow conditions, and the method used to quantify the mucus barrier.

**Fig. 4.**
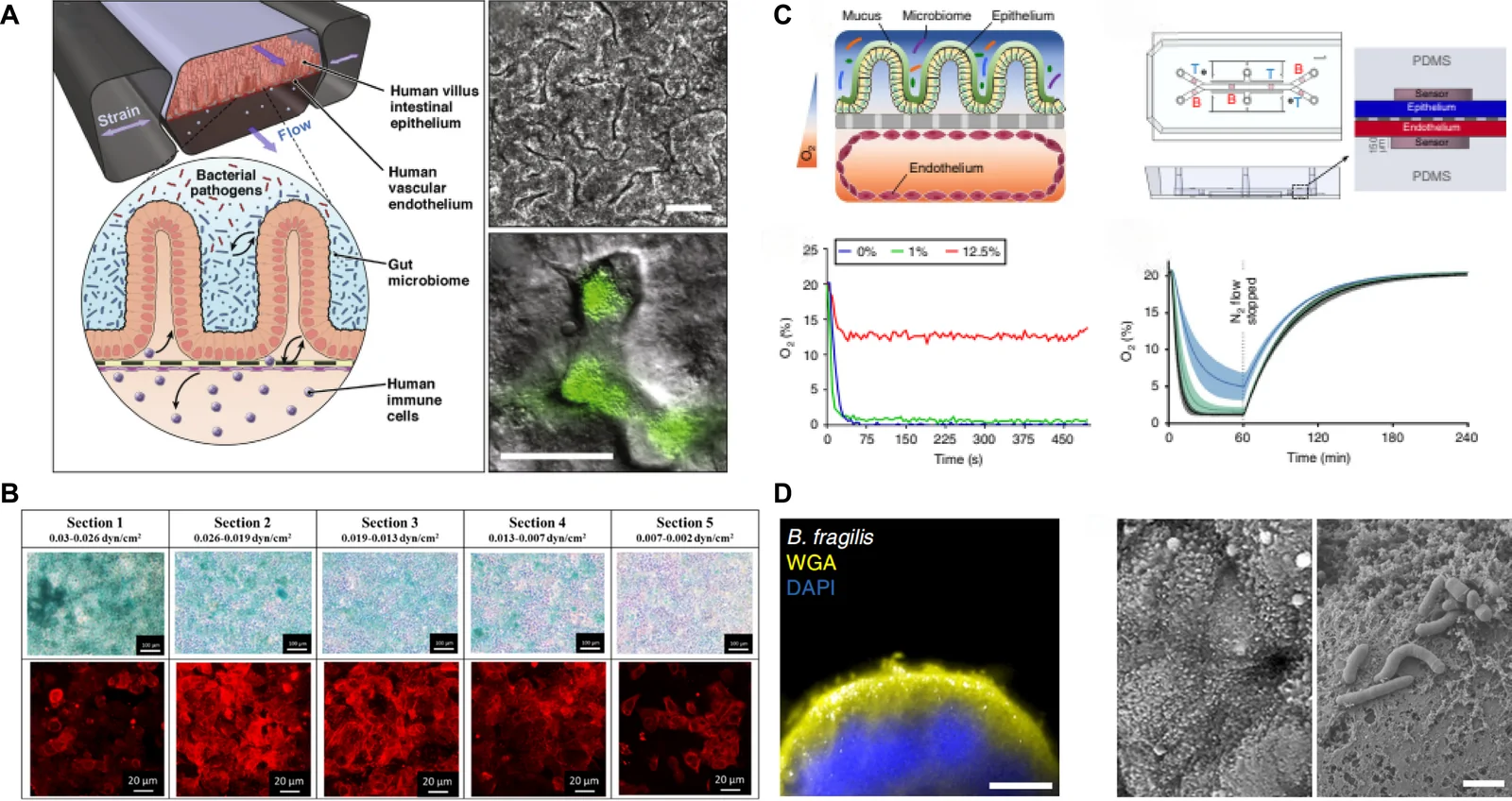
Simulation of mucus secretion in gut-on-a-chip systems and factors that influence it [2,40,53]. (A) The gut chip forms villus-like structures and produces mucus during coculture with living commensal microbes [2]. The overlaid image shows coculture of green fluorescent protein-labeled *E. coli* with microengineered villi in the gut chip. Scale bars, 50 μm. (B) Mucus production by Caco-2 monolayers under different shear-stress conditions. MUC2 expression was lower under near-static conditions and higher under intermediate fluid shear stress [53]. (C) Oxygen-sensitive human intestine chip with an oxygen gradient [40]. (D) Mucus layer formed between the epithelium and cocultured commensal microorganisms (*Bacteroides fragilis*) in the intestine chip [40]. Left, cross-sectional fluorescence microscopy of the Caco-2 epithelium, mucus layer, and bacteria; right, scanning electron microscopy of the apical epithelial surface. Scale bars are indicated in the original figure.

## Application of Gut-on-a-Chip in Modeling Pathogen Infections

Gut-on-a-chip platforms can reproduce selected environmental features that are important for intestinal infection, including flow, epithelial polarity, oxygen gradients, mucus, and compartmentalized host–microbial interactions [30,40,58]. Available evidence is uneven across pathogen classes: bacterial applications are comparatively mature [3,30,46,48,55], whereas viral and parasitic studies are more heterogeneous and fungal applications remain sparse [13,59–62] (see Table 4). Accordingly, the following sections distinguish established mechanistic demonstrations from emerging or conceptually plausible applications.

**Table 4.** Relative evidence maturity of gut-on-a-chip applications across pathogen classes

Pathogen class	Representative examples	Current evidence maturity a	Most defensible use of gut-on-a-chip	Main unresolved issues
Bacteria	*Shigella*, *Salmonella*, pathogenic *E. coli*, *C. difficile*, probiotic–pathogen interactions	Relatively mature within the intestinal infection field	Mechanistic studies of adhesion/invasion, barrier injury, colonization, toxin effects, immune interactions	Need standardized conditions, broader donor diversity, and more head-to-head benchmarking
Viruses	Norovirus, SARS-CoV-2, rotavirus	Intermediate and heterogeneous	Viral tropism, replication dynamics, barrier responses, innate immune signaling	Smaller evidence base, pathogen-specific receptor/medium requirements, limited clinical benchmarking
Parasites	Cryptosporidium, Entamoeba	Early to intermediate; model-dependent	Persistent epithelial interaction, tissue injury, parasite life-cycle-related questions	Long-term culture, parasite-specific biology, throughput, and limited comparative validation
Fungi	Direct intestinal gut-on-a-chip evidence is limited in the current literature set	Limited/emerging	Potential future use for epithelial invasion, barrier injury, and host–fungal–microbiome interactions	Insufficient direct evidence to support strong claims of maturity or translational readiness

^a^ Evidence maturity is a qualitative synthesis of the scope and validation represented in the cited literature; it is not a quantitative meta-analysis.

### Bacterial infection studies

Bacterial pathogens currently represent the broadest gut-on-a-chip evidence base. Studies of *Salmonella*, *Shigella*, *Clostridioides difficile*, pathogenic *Escherichia coli*, and probiotic–pathogen interactions have used chips to examine adhesion, invasion, toxin-mediated injury, barrier disruption, immune recruitment, colonization under flow, and host–microbiome interactions [3,14,46,55,63–65] (Fig. 5). The most informative use of a chip is obtained when dynamic flow, mechanical stimulation, mucus, oxygen gradients, or cross-compartment immune interactions alter an infection phenotype that is not readily accessible in a static monolayer. These studies establish mechanistic utility, but they do not by themselves demonstrate superior prediction of human clinical outcomes.

**Fig. 5.**
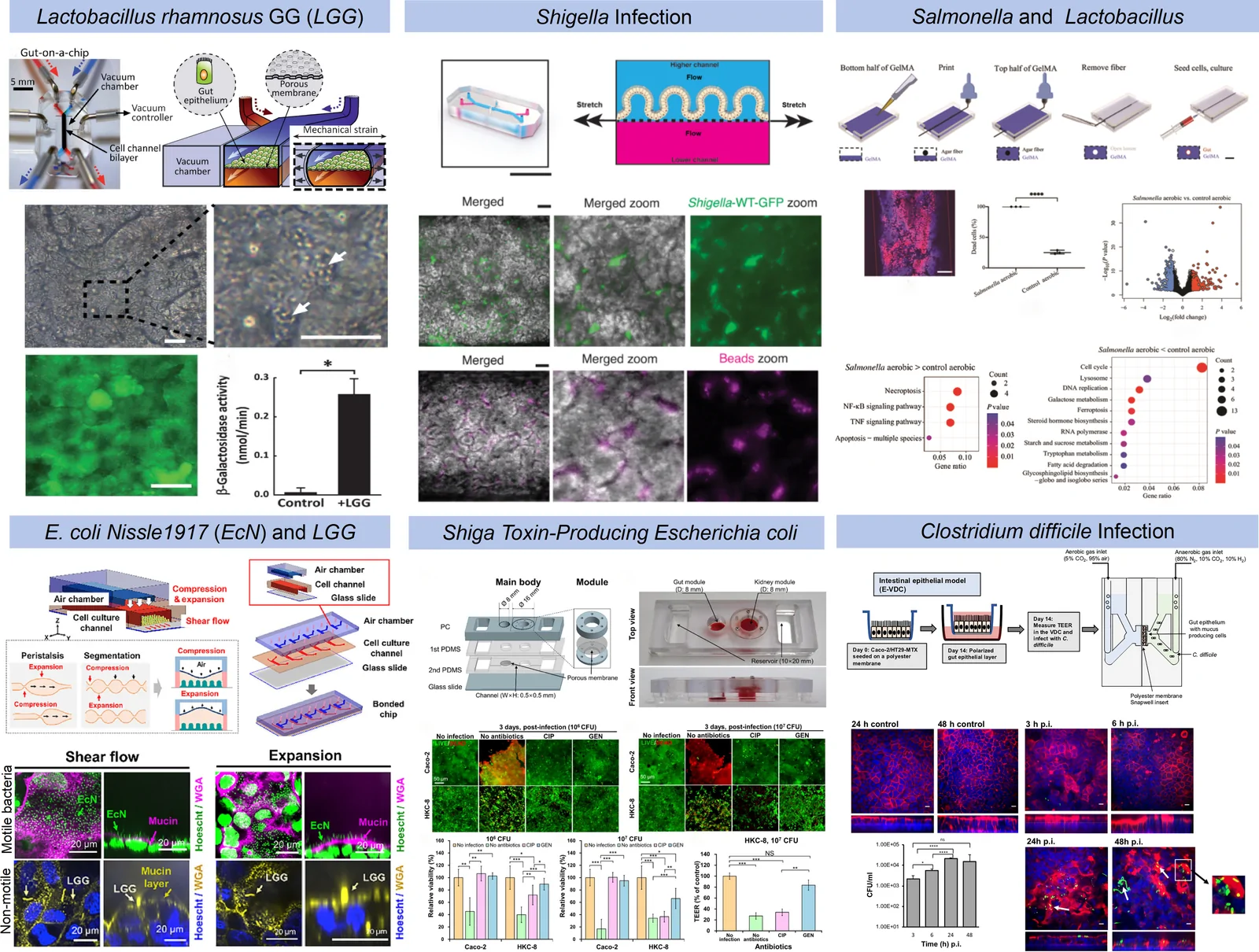
Representative gut-on-a-chip models used to study bacterial infection. Examples include *Lactobacillus rhamnosus GG* (*LGG*) [30], *E. coli Nissle 1917* (*EcN*) and *LGG* [63], *Shigella* infection [55], Shiga toxin-producing *E. coli* [64], *Salmonella* and *Lactobacillus* [65], and *Clostridium difficile* infection [46].

### Viral and parasitic infection studies

Evidence for viral and parasitic infection models is growing but remains narrower than the bacterial literature. Human intestinal enteroid-based systems have enabled productive norovirus infection studies [60,61]. ACE2-expressing intestinal chips have been used to characterize SARS-CoV-2 replication and epithelial and interferon responses [13,62], while rotavirus models have examined tight-junction disruption [66]. For parasites, chip and related microfluidic systems have supported studies of *Cryptosporidium* and *Entamoeba*, including epithelial injury and parasite persistence [59,67,68]. These examples demonstrate feasibility and mechanistic value, but study numbers, platform architectures, and validation depth remain heterogeneous. A 2025 gut-on-a-chip study extended in vitro development of *Cryptosporidium hominis* under defined fluid shear, strengthening the evidence base for parasitic infection while also illustrating the need for pathogen-specific optimization [69]. A 2026 intestinal microphysiological-system study further demonstrated persistent enterovirus A71 infection for up to 14 d without overt epithelial injury, providing a useful example of how sustained infection does not necessarily reproduce severe tissue damage [70]. Key environmental parameters and measurable phenotypes for different types of microbial infection in gut-on-a-chip studies are summarized in Table 5.

**Table 5.** Key environmental parameters and measurable phenotypes for different microbial infection classes in gut-on-a-chip studies

Bacteria type/name	Key simulated external environment	Chip implementation strategy and parameters (based on previous research)	Main research objectives/phenotypes	Representative references
Facultative anaerobes Representative: *E. coli* (EHEC/EPEC), *Salmonella*	1. Oxygen gradient	Create a hypoxic environment at the apical side (epithelial layer) and maintain a normoxic environment at the basolateral side (“vascular” channel) through channel design; verify using hypoxia indicators or oxygen sensors.	Reproduce survival under gut-like conditions; study competition/coexistence with obligate anaerobes; induce virulence gene	[40]
	2. Fluid shear stress	Apply low-flow-rate medium perfusion to simulate intestinal mucus and content flow.	Enhance bacterial colonization and biofilm formation; affect bacterial gene expression profiles.	[30,55]
	3. Mechanical deformation	Simulate intestinal peristalsis by applying cyclic stretching to the porous membrane via vacuum suction in side chambers.	Promote host cell differentiation, forming a barrier closer to in vivo conditions, affecting host–pathogen interactions; may influence pathogen colonization strategies on dynamic surfaces.	[30,55]
	4. Mucus layer	Coculture with goblet cells (e.g., HT29-MTX) or add purified mucus to the exposed apical surface to form an in vivo-like, stratified mucus barrier.	Study how pathogens penetrate the mucus layer; assess their damage to mucus degradation and barrier integrity.	[3,57,96]
	5. Immune cell recruitment	Introduce immune cells into the basolateral channel; natural recruitment via chemokines produced by infected epithelial cells, or direct injection.	Visualize real-time immune response; study bacterial mechanisms of evading host immune clearance; simulate infectious inflammation.	[14]
Obligate anaerobes Representative: *Bacteroides spp*.; *Clostridium spp*.	1. Strict hypoxic environment	Establish and maintain a stable hypoxic zone at the apical side, using anaerobic workstations for chip pretreatment and inoculation, or integrating onboard oxygen scavenging systems.	Enable long-term, stable coculture with human gut microbiome; study microbial diversity, metabolic interactions, and colonization resistance.	[105]
	2. Microbial community niche	Sequential or mixed inoculation of multiple bacterial species, providing complex media to support diverse metabolic needs.	Evaluate their role in colonization resistance against pathogens; study the impact of microbial metabolites (e.g., SCFAs) on host health.	[105]
	3. Host–microbe interaction	Establish anaerobic communities on differentiated, functional gut epithelium.	Study how commensal bacteria affect host barrier function, immune tolerance, and energy metabolism.	[46,96,105]
Invasive pathogens Representative: *Shigella* spp.	1. Polarized epithelium and M cells	Culture epithelial monolayers with complete polarity.	M cells can serve as an invasion route for pathogens such as *Salmonella* and *Shigella*; study their adhesion, internalization, and transcytosis mechanisms.	[55]
		Induce M cell differentiation through coculture or adding signaling factors (e.g., RANKL).		
	2. Basolateral contact and immune evasion	Real-time, high-resolution imaging to track bacteria from apical invasion, through cells, to the basolateral space.	Simulate the initial steps of bacterial dissemination from the intestinal lumen to the systemic circulation; study their intracellular survival and replication.	[55]
	3. Strong inflammatory response	Real-time monitoring of epithelial barrier function decline, pro-inflammatory cytokine secretion, and immune cell infiltration.	Recapitulate the acute inflammatory pathology of bacterial dysentery; evaluate anti-inflammatory therapies.	[55]
Viruses Representative: norovirus; rotavirus; enterovirus A71; SARS-CoV-2	1. Functional host cell receptors	Use highly differentiated human intestinal epithelial cells fully expressing apical receptors (e.g., glycosylated HBGAs for norovirus).	Enable robust replication of hard-to-culture viruses, unachievable with traditional cell lines.	[38,60,61]
	2. Bile acid environment	Add physiological concentrations of bile acids to the flowing medium.	May be required for efficient replication of some viruses (e.g., norovirus).	[38,106]
	3. Dynamic mucus layer	Establish a functional mucus layer to study viral penetration of this physicochemical barrier.	Evaluate virus-mucus component interactions and the role of neutralizing antibodies at the mucosal surface.	[13,107]
	4. Innate immune response	Simulate virus-triggered type I and III interferon responses by coculturing immune cells.	Study viral immune evasion mechanisms and how host immunity limits or clears infection.	[62,70,108,109]
Parasites Representative: *Cryptosporidium*; *Entamoeba histolytica*	1. 3D villus structure	The 3D villus structures formed in the chip provide physical anchoring sites for apical parasitism (e.g., Cryptosporidium).	Simulate their unique intracellular but extra-cytoplasmic parasitic niche; study their life cycle.	[59,68,69]
	2. Fluid shear stress	Continuous fluid shear stress simulating intestinal content flow, crucial for oocyst detachment and transmission.	Study how parasites resist fluid washout to maintain infection, and transmission dynamics.	[59]
	3. Tissue invasion and destruction	Real-time imaging to track how trophozoites (e.g., *E. histolytica*) contact, kill, and penetrate epithelial cells, causing characteristic “flask-shaped” ulcers.	Visualize and quantify epithelial barrier disruption; test anti-parasitic drugs.	[67]

### Fungal infection models

Although less mature than bacterial and viral applications, recent gut-on-a-chip studies have demonstrated the feasibility of modeling *Candida albicans* colonization, epithelial barrier disruption, fungal translocation, immune responses, and antifungal treatment under dynamic microphysiological conditions [71,72]. These studies support inclusion of fungi as an emerging application area rather than as an established infection-model category.

## Advanced Models: Host–Microbiome Interactions and Infection-Relevant Gut–Organ Axes

### Molecular mechanisms of host–microbiome interactions

Gut-on-a-chip models can be particularly useful for host–microbiome questions when flow, oxygen partitioning, mucus, and epithelial differentiation influence microbial persistence or host responses [40,73]. In infection settings, these features can be used to test whether commensal communities or microbial metabolites alter pathogen colonization, epithelial barrier function, inflammatory signaling, or therapeutic response. Because current multi-species communities remain much simpler than the native microbiome, findings should be interpreted as controlled ecological experiments rather than complete reproductions of the human gut microbiota. Recent consensus work on microbiome causality likewise emphasizes donor variability, representativeness, standardization, and the need to combine complementary models rather than treating a single in vitro platform as sufficient evidence of causality [74].

### Infection-relevant gut–organ axis on a chip

Gut–organ-axis systems are relevant to infectious disease when an intestinal event is hypothesized to alter a distal organ through circulating pathogens, microbial products, immune mediators, or drug exposure (Fig. 6 and Table 6). However, many published gut–brain [75–79], gut–liver [80–82], gut–lung [83–85], gut–skin [86–89], gut–kidney [64,90], and gut–islet [91] examples primarily address metabolism, toxicity, inflammation, or noninfectious physiology rather than infection-specific validation. Notable exceptions exist—for instance, gut–brain translocation of *E. coli* K1 [79], Shiga toxin-producing *E. coli*-mediated kidney injury [64], and hepatitis E virus transmission in a gut–liver platform [80]—but these remain isolated demonstrations rather than a mature body of evidence. In this review, these platforms are therefore treated as enabling architectures unless a study directly links an intestinal infection to a distal-organ phenotype.

**Fig. 6.**
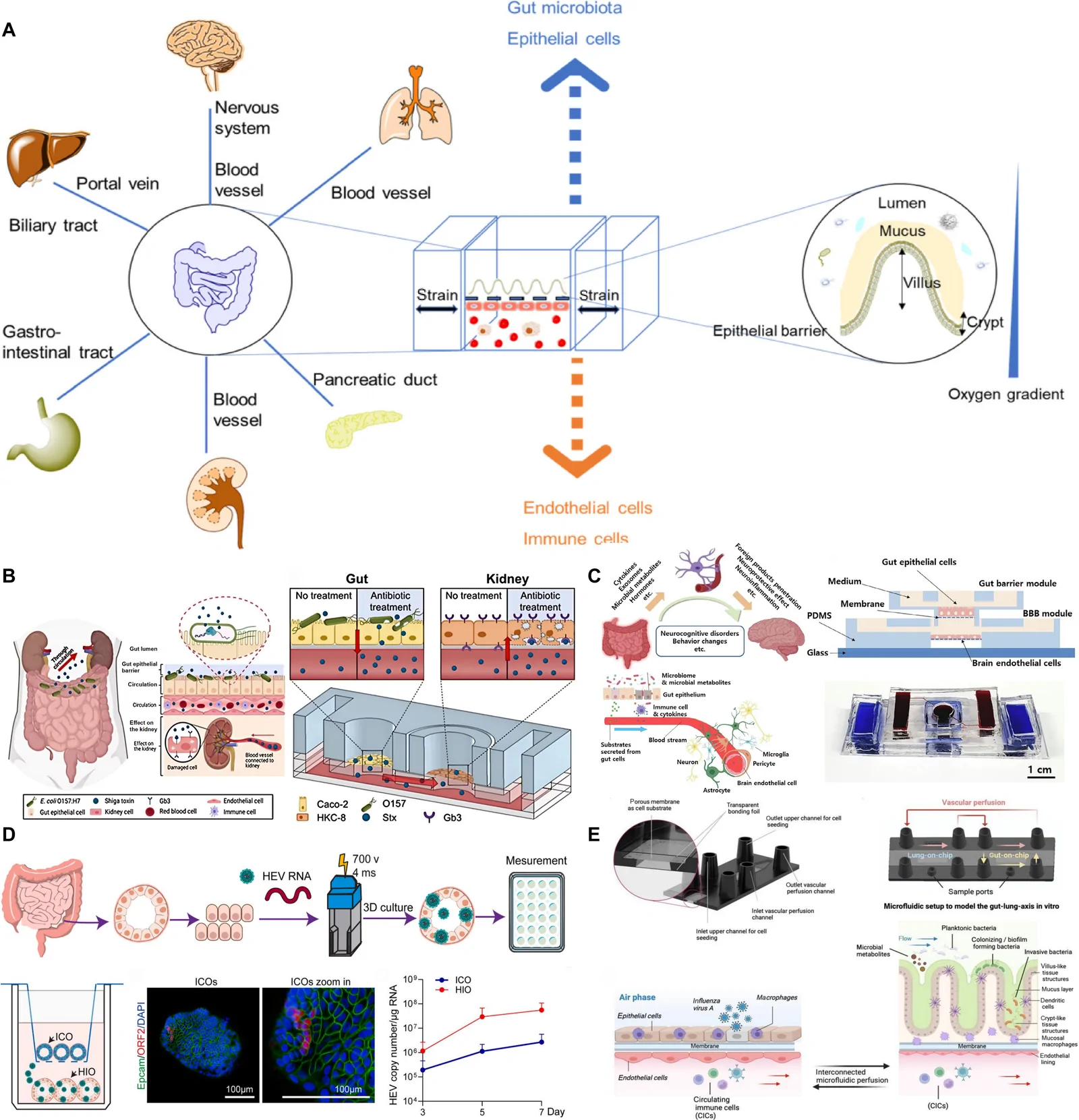
Representative gut-organ-axis-on-chip architectures. (A) Schematic diagram of the organoids/organs-on-a-chip model [97]. (B to D) Infection- or host-response-relevant examples, including the gut–kidney axis [64] (B), gut–brain axis [78] (C), and gut–liver axis [80] (D). (E) Conceptual extrapolation relevant to the development of the intestinal–lung axis platform [85].

**Table 6.** Overview of gut axis on chip model studies of different types. PAMP: pathogen-associated molecular pattern.

Gut–organ axis type	Cell types used	Chip type and connection method	Model key features	Main research objectives	Representative studies and other key refs.
Gut–brain axis (established infection)	Gut: Caco-2/intestinal organoid-derived epithelium; Brain: iPSC-derived neurons, astrocytes, microglia; BBB: brain microvascular endothelial cells	Common type: Modular multi-chamber chip containing gut, BBB, and neuron regions. Connection: Connected via a shared “vascular” channel.	Focus on the impact of gut microbiome metabolites on BBB and neuronal function	Exploring gut microbiome-mental health links	Rep: [79] Other: [75–79]
Gut–kidney axis (established infection)	Gut end: Caco-2. Kidney end: Human renal proximal tubule epithelial cells (HK-2), human kidney cortical epithelial cells (HKC-8)	Common type: Tandem multi-chamber chip. Connection: Effluent from gut chamber serves as input for kidney chamber.	Study the clearance function of the kidney for uremic toxins produced by gut microbiome metabolism.	Chronic kidney disease mechanisms	Rep: [64,110] Other: [90]
Gut–liver axis (PAMP/inflammation)	Gut end: Caco-2, human primary intestinal stem cell-derived organoids. Liver end: Human hepatoma cells (HepG2, HepaRG), human primary hepatocytes.	Common type: Multi-chamber microfluidic chip connected in series via microchannels. Connection: Effluent from gut chamber directly perfused into liver chamber.	Simulate the effects of gut microbiome metabolites on hepatocytes; investigate substance exchange in the portal vein circulation.	Enterohepatic circulation research	Rep: [82] Other: [80–82]
Gut–lung axis (conceptual extrapolation)	Gut end: Caco-2, human primary intestinal organoids. Lung end: Human lung adenocarcinoma cells (A549/HBEpic), macrophages	Common type: Multi-chamber platform with gut and lung epithelial modules. Connection: Connected via a shared “vascular” circulating medium.	Study how gut microbiome metabolites regulate distal lung immunity via circulation; simulate the common mucosal immune system; explore the impact of gut inflammation on lung disease susceptibility.	Exploring lung infection–gut microbiome links	Rep: [85] Other: [83–85]
Gut–skin axis (conceptual extrapolation	Gut end: Caco-2. Skin end: Human immortalized keratinocytes (HaCaT), human primary keratinocytes/fibroblasts coculture (artificial skin model).	Common type: Multi-organ chip platform integrating gut and skin modules. Connection: Connected via a common circulating medium.	Investigate how gut-derived metabolites and immune regulators affect skin tissue; focus on gut microbiome-diet interactions.	Inflammatory skin disease mechanisms	Rep: [86] Other: [86–89]

## Current Challenges and Future Perspectives

### Persistent technical limitations

Despite rapid technical progress, several barriers limit reproducibility and translational interpretation. Functional stability may decline during prolonged culture, especially when epithelial state, mucus, or microbial composition changes [92,93]. Microbial communities in current chip studies are usually simplified relative to the in vivo ecosystem [47]. Standardization remains a major issue because results can depend on cell source, donor characteristics, matrix or membrane composition, chip geometry and material, flow and strain parameters, oxygen gradients, microbial inoculum and multiplicity of infection (MOI), barrier measurements such as transepithelial electrical resistance (TEER), sampling time, and endpoint definitions. Throughput is also generally lower and operational complexity higher than in conventional 2D assays [92,94]. Recent studies further demonstrate that mucus and microbial biofilms can materially alter gut-on-a-chip phenotypes, supporting the inclusion of mucus source, thickness, microbial composition, and flow conditions in minimum reporting requirements [92].

### From demonstration to validation

Future progress should prioritize validation rather than complexity alone. Multi-organ integration can be valuable when a defined infection mechanism requires systemic communication, but near-term work should emphasize controlled, infection-specific questions and benchmarked endpoints. Patient-derived cells may support individualized susceptibility or therapeutic-response studies, yet such applications require prospective comparison with patient phenotypes or clinical outcomes. Artificial intelligence (AI) may improve image-based phenotyping, quality control, segmentation, or multimodal data integration, but direct evidence that AI has already improved gut-on-a-chip infectious-disease prediction remains limited. Accordingly, AI is best framed as an emerging analytical layer rather than an established determinant of translational performance. For AI specifically, recent work has demonstrated supervised-learning-based quality assessment of organ-on-a-chip microscopy images, but this evidence comes mainly from noninfectious chip-quality tasks rather than direct prediction of gut infection outcomes [95].

### Minimum reporting and quality-control priorities

To improve reproducibility, infection studies should report a minimum set of biological, engineering, microbiological, and analytical variables. Table 7 provides a practical reporting checklist that can be adapted to the pathogen and chip architecture.

**Table 7.** Recommended reporting checklist for gut-on-a-chip infection studies

Reporting domain	Minimum information to report	Why it matters for infection studies
Biological source	Cell type, species, donor age/sex when available, disease status where relevant, passage number	Determines receptor expression, susceptibility, and inter-donor variability
Cell phenotype	Differentiation protocol; epithelial, endothelial, immune, or stem-cell state; key markers	Allows comparison of infection competence and barrier phenotype
Matrix/membrane	ECM identity, coating method, concentration, membrane material, pore size, thickness	Affects adhesion, diffusion, cell phenotype, and pathogen transport
Chip geometry/materials	Channel dimensions, membrane arrangement, material (e.g., PDMS), bonding and surface treatment	Controls transport, adsorption, shear, oxygen transfer, and device reproducibility
Physical cues	Flow rate or calculated shear stress; strain amplitude/frequency; pressure; oxygen level or gradient	Provides the mechanical and chemical boundary conditions for infection
Microbial inoculation	Strain/isolate, growth phase, inoculum size, MOI where applicable, exposure route, antibiotic pretreatment	Essential for interpreting pathogen kinetics and inter-study differences
Barrier/infection endpoints	TEER method and normalization, permeability tracer, pathogen load, replication assay, imaging, cytokines, cell-death metrics	Prevents ambiguous or noncomparable outcome definitions
Sampling/time	Baseline, infection, intervention, and sampling time points; duration and dropout criteria	Required for kinetic interpretation and long-term stability assessment
Data/statistics	Biological versus technical replicates, randomization/blinding where feasible, statistical model, exclusion criteria, raw-data availability	Distinguishes device variability from biological effects and supports reproducibility

### Validation and translational readiness

A practical validation ladder is preferable to a single claim of “physiological relevance”. Construct validity asks whether the chip reproduces the intended tissue feature; mechanistic validity asks whether it reproduces a defined infection mechanism; comparative validity asks whether it adds predictive or mechanistic value over existing in vitro or organoid models; and translational validity asks whether the model predicts patient observations, therapeutic response, toxicity, or other clinically relevant outcomes (Table 8). Recent advances in intestinal organ-chip standardization emphasize the importance of predefined requirements for cell sources, device architecture, mechanical stimulation, functional readouts, and validation strategies to improve reproducibility and translational relevance [24]. However, available gut-on-a-chip infection studies are concentrated in the first 2 levels, with fewer studies providing head-to-head comparative or clinical benchmarking [1]. Claims about precision medicine and animal replacement should therefore be matched to the highest validation level actually demonstrated.

**Table 8.** A perspective on the validation ladder for gut-on-a-chip infection research

Validation level	Core question	Evidence that would support the claim	Current interpretation for gut-on-a-chip infection research
1. Construct validity	Does the model reproduce the intended intestinal feature?	Barrier integrity, polarity, mucus, flow/strain response, oxygen gradient, cell composition	Commonly demonstrated
2. Mechanistic validity	Does it reproduce a defined host–pathogen mechanism?	Pathogen replication/invasion, toxin effects, immune signaling, microbiome-mediated effects	Increasingly demonstrated, especially for bacterial infections
3. Comparative validity	Does it add predictive/mechanistic value over simpler models?	Prospective head-to-head comparison against 2D, organoid, or animal models using matched endpoints	Still limited and uneven across pathogens
4. Translational validity	Does it predict clinically relevant human outcomes?	Concordance with patient phenotypes, drug responses, toxicity, or prospective clinical datasets	Sparse; currently a major evidence gap rather than an established capability

## Conclusion and Outlook

Gut-on-a-chip systems provide a controllable, human-relevant in vitro environment for selected intestinal infection questions. Their principal value is not that they universally surpass simpler models, but that they can introduce specific features—such as flow, mechanical deformation, mucus, oxygen gradients, endothelial or immune compartments, and controlled microbiota—that are difficult to combine in conventional assays. The evidence base is most developed for mechanistic bacterial studies, whereas viral and parasitic applications remain more heterogeneous and direct fungal applications are limited.

The major barriers to broader use are therefore not only technical complexity but also reproducibility, standardized reporting, microbial stability, throughput, and validation against established models and human observations. Future studies should prioritize pathogen-specific benchmarking, explicit reporting of key engineering and infection parameters, and comparative or prospective validation. We propose an evidence-based framework for gut-on-a-chip models in infectious diseases, spanning fundamental biological questions to translational perspectives (Fig. 7). In addition, AI, 3D bioprinting, patient-derived cells, and multi-organ integration are promising enabling technologies, but their contribution should be judged by whether they improve measurable prediction or mechanistic insight rather than by complexity alone.

**Fig. 7.**
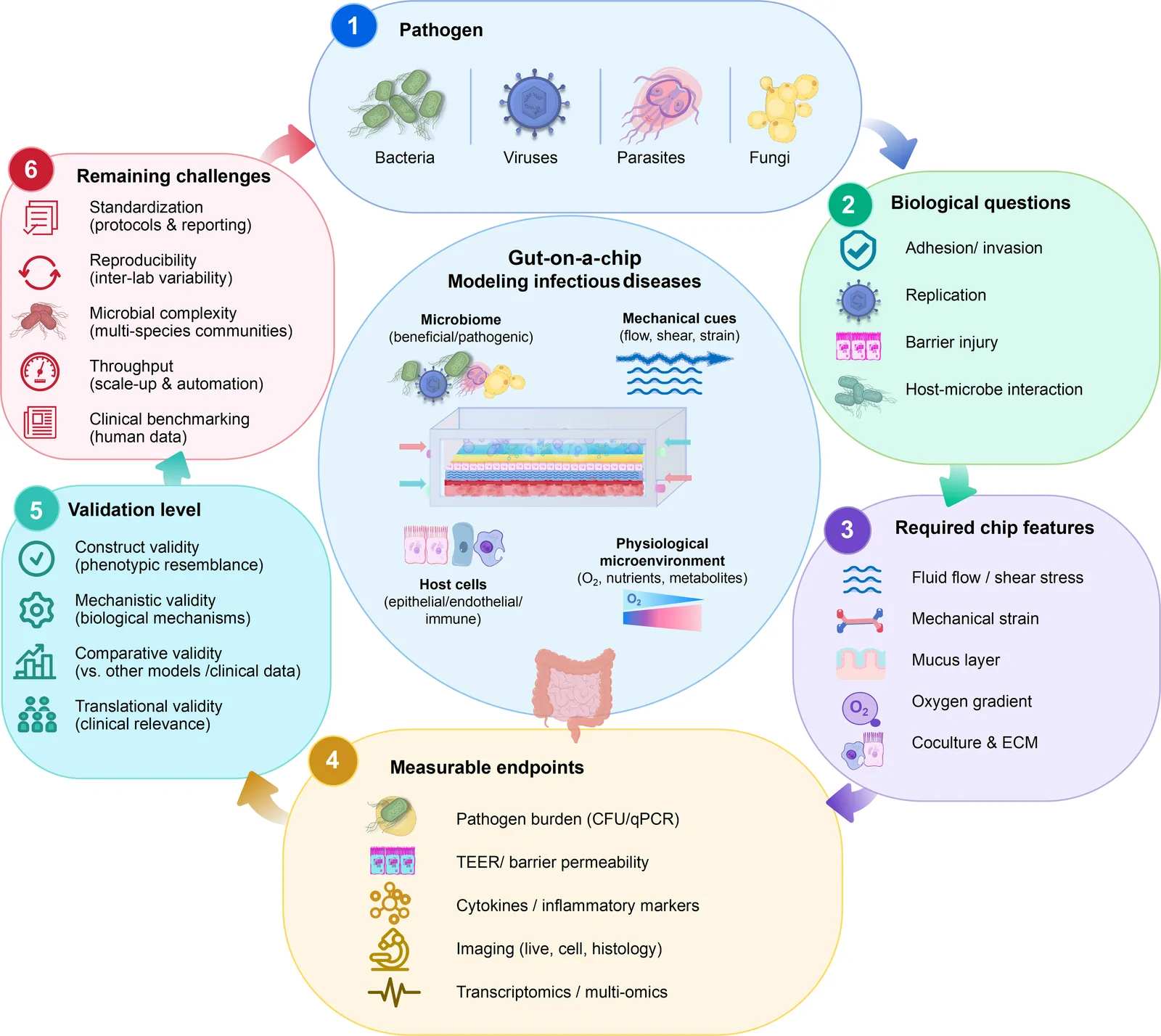
An evidence-based framework from biological questions to translational perspectives for gut-on-a-chip modeling of infectious diseases. Created using WPS; some icons were generated using the CNSknowall platform (https://cnsknowall.com).
